# Using Machine Learning for the Risk Factors Classification of Glycemic Control in Type 2 Diabetes Mellitus

**DOI:** 10.3390/healthcare11081141

**Published:** 2023-04-15

**Authors:** Yi-Ling Cheng, Ying-Ru Wu, Kun-Der Lin, Chun-Hung Richard Lin, I-Mei Lin

**Affiliations:** 1 Department of Psychology, College of Humanities and Social Sciences, Kaohsiung Medical University, Kaohsiung 807378, Taiwan; yilingcheng@kmu.edu.tw (Y.-L.C.); skywuu22@gmail.com (Y.-R.W.); 2 The Lin’s Clinic, Kaohsiung 807057, Taiwan; bergkmu@gmail.com; 3 Department of Computer Science and Engineering, National Sun Yat-sen University, Kaohsiung 80424, Taiwan; lin@cse.nsysu.edu.tw; 4 Department of Medical Research, Kaohsiung Medical University Hospital, Kaohsiung 807378, Taiwan

**Keywords:** artificial intelligence, depression, glycemic control, machine learning, type 2 diabetes mellitus

## Abstract

Several risk factors are related to glycemic control in patients with type 2 diabetes mellitus (T2DM), including demographics, medical conditions, negative emotions, lipid profiles, and heart rate variability (HRV; to present cardiac autonomic activity). The interactions between these risk factors remain unclear. This study aimed to use machine learning methods of artificial intelligence to explore the relationships between various risk factors and glycemic control in T2DM patients. The study utilized a database from Lin et al. (2022) that included 647 T2DM patients. Regression tree analysis was conducted to identify the interactions among risk factors that contribute to glycated hemoglobin (HbA1c) values, and various machine learning methods were compared for their accuracy in classifying T2DM patients. The results of the regression tree analysis revealed that high depression scores may be a risk factor in one subgroup but not in others. When comparing different machine learning classification methods, the random forest algorithm emerged as the best-performing method with a small set of features. Specifically, the random forest algorithm achieved 84% accuracy, 95% area under the curve (AUC), 77% sensitivity, and 91% specificity. Using machine learning methods can provide significant value in accurately classifying patients with T2DM when considering depression as a risk factor.

## 1. Introduction

The prevalence of diabetes mellitus was 10.5% among individuals aged 20–79 years in 2021, and it is expected to increase to 12.2% by 2045. Type 2 diabetes mellitus (T2DM) accounts for the largest portion of DM cases. The global diabetes-related health expenditures were about 966 billion USD in 2021 and are estimated to reach 1054 billion USD by 2045 [1]. Regarding the risk factors for T2DM, Ismail et al. [2] reviewed 106 studies and found that high-level serum uric acid, sleep quality and quantity, smoking, depression, cardiovascular disease, dyslipidemia, hypertension, aging, ethnicity, family history of diabetes, physical inactivity, and obesity were related to development of T2DM. Haghighatpanah et al. [3] found that female patients who were aged younger than 65 years old, obese (body mass index [BMI] ≥ 30), engaging in housework, with low high-density lipoprotein (HDL) level, and on certain types of medication were more likely to have poor glycemic control in patients with T2DM and had secondary medical complications.

Poor glycemic control was related to sociodemographic factors (such as duration of diabetes, age of onset, family history, job status, educational status, etc.), medical status (hypertension, lipid profiles, and fasting plasma glucose levels), lifestyle (dietary compliance, physical activity, self-blood glucose monitoring, and drug compliance), and complications [4]. Research also found that profiles with high lipid levels (such as low-density lipoprotein [LDL]), LDL/HDL ratio, and triglycerides [TG]/HDL ratio) were predictive markers for poor glycemic control in T2DM [5]. Poor glycemic control is associated with increased hypoglycemia, cardiovascular disease, sudden death during a severe episode, and mortality in diabetes [6]. Therefore, defining the risk factors for poor glycemic control in T2DM is important for preventing poor prognosis and for medical management.

Studies utilizing machine learning methods to predict diagnostic outcomes for chronic diseases have become increasingly popular [7,8]. In the case of diabetes, numerous studies have compared the effectiveness of various machine learning methods [9,10,11,12,13,14,15]. Nusinovici et al. [16] conducted a study that examined four different chronic diseases (including diabetes, hypertension, cardiovascular diseases, and chronic kidney disease) using machine learning techniques, such as random forest, neural networks, and standard logistic regression. Of these diseases, diabetes prediction is of significant interest. For instance, Dagliati et al. [17] identified logistic regression with machine learning as a useful tool for predicting factors related to different diabetes complications, such as retinopathy, neuropathy, and nephropathy at different time points using longitudinal data. Machine learning has become popular in predicting the probability of having diabetes, and many studies have used different variables or attributes, such as background information, BMI, and heart rate [9,10,11,12,13,14,15]. These studies used machine learning approaches to classify the likelihood of having diabetes based on these variables. Research into diabetes has led to the development of risk factor scores that enable patients to assess their own risk factors and self-care needs. For instance, Lindstrom and Tuomilehto [18] proposed a method of summing risk factor scores based on factors like physical activity and fruit and vegetable consumption. Bang et al. [19] developed a new score using several individual scores, including obesity and health habits. Recently, Yang et al. [20] went a step further by using big data and creating an online risk factor score calculation system for personalized health management. While these studies have been effective in detecting type 2 diabetes mellitus in a non-invasive and cost-effective way, they have one limitation: emotional factors have not been incorporated into the risk score calculation. The popularity of machine learning methods can be seen in the increasing number of review papers published in recent years, including those by Kavakiotis et al. [21], Abhari et al. [22], and Olusanya et al. [23]. While previous studies have shown promising results in applying machine learning methods to diabetes research, negative emotions (such as depression and anxiety) have received less attention. Some studies have investigated the use of machine learning methods to predict anxiety or depression [24,25], and satisfactory prediction rates have been achieved. However, only a few studies have examined negative emotions with diabetes-related conditions [26,27,28], and research is scarce in this direction. Ducat et al. [29] reviewed the literature and identified anxiety, depression, and eating disorders as the three major health comorbidities for diabetes.

The current state of research suggests that negative emotions (such as anxiety and depression) have not been fully considered in machine learning applications for predicting diabetes, despite evidence suggesting a connection between these factors and diabetes [30,31,32]. The careful selection of the variables from relevant literature is critical for enhancing the model’s predictive power [33,34]. Therefore, further research is needed to deepen our understanding of these relationships and contribute to the field. To address this gap, the present study aims to explore the potential interactions of risk factors using regression tree analysis and to identify the best machine learning method for the classification of diabetes when negative emotions are involved. It is hoped that this study will contribute to the development of more accurate and effective prediction models for diabetes, considering the impact of negative emotions.

## 2. Materials and Methods

### 2.1. Participants

Participants were recruited from the Department of Internal Medicine at Kaohsiung Medical University Hospital and Kaohsiung Municipal Siaogang Hospital. A total of 647 patients aged over 20 years old and diagnosed with T2DM completed the study between 25 August 2020 and 30 May 2021. The original data used traditional statistical analysis to explore the association between cardiac autonomic activity and glycemic control in T2DM and was published by Lin et al. [35]. There were 361 males (56%) and 286 females (44%). Participants’ mean age was 62.64 (SD = 10.32, range from 31 to 91 years old). The mean of the HbA1c value was 7.25% with an SD of 1.18%, ranging from 4.68 to 15.13%); the mean and SD of depression were 2.10 and 3.02, respectfully, with 84.70% within the normal range and 15.30% higher than mild depression; and the mean and SD of anxiety were 1.38 and 2.46, respectfully, with 90.88 % within the normal range and 9.12% higher than mild anxiety.

This study used machine learning of artificial intelligence to figure out the best prediction model for multiple risk factors in glycemic control. Patients with a pacemaker or arrhythmia were excluded since these conditions cannot correctly analyze the HRV indices. The institutional review board was approved by the ethics committee of Kaohsiung Medical University Hospital (KMUHIRB-E(I)-20200194), and informed consent was obtained from each patient before the study.

### 2.2. Measurement

Demographic data, including age, gender, and BMI, were collected. All participants were measured with the Patient Health Questionnaire-9 (PHQ-9) [36] and Generalized Anxiety Disorder-7 (GAD-7) [37] for the symptoms of depression and anxiety. The PHQ-9 includes nine items with a four-point Likert scale ranging from 0 to 3 to measure depressive symptoms during the past two weeks. The internal consistency (Cronbach’s α) of the PHQ-9 was 0.86 to 0.89, and the test-retest reliability was 0.84 [38]. The GAD-7 includes seven items with a four-point Likert scale ranging from 0 to 3 to measure anxiety-related symptoms during the past two weeks. The internal consistency (Cronbach’s α) of the GAD-7 was 0.92 and the intraclass test-retest reliability was 0.83 [37].

Subsequently, the electrocardiography (ECG) signals were collected by using a lead II QOCA portable ECG monitoring device (Quanta Computer Inc., Taiwan), which was approved by the Ministry of Health and Welfare, Taiwan (Number 005428). The five-minute ECG was measured for each patient at a sitting and resting baseline. The ECG device was connected to a Samsung Galaxy Tab A 10.1 SM-T515 (Samsung, Gyeonggi-do, Republic of Korea), which raw ECG signals that were then uploaded to the QOCA platform (Quanta Computer Inc., Taoyuan, Taiwan).

Blood samples included the lipid profiles (HDL, LDL, and TG) and HbA1c and were obtained from the electronic medical records system during the three months since the ECG had been measured. All the blood samples were measured at at least 12 h fasting.

### 2.3. Data Reduction and Statistical Analysis

Researchers checked the ECG waveform and deleted arrhythmia and movement artifacts. The interbeat interval (IBI) data were downloaded from the QOCA platform through Python software (Quanta, Taiwan) and then imported to the CardioPro Infiniti HRV Analysis Module (Thought Technology Ltd., Montreal, Quebec, Canada), which transformed the IBI data into the time and frequency domains of HRV. The time domain of HRV included standard deviation of normal-to-normal intervals (SDNN), root mean square of the successive differences (RMSSD), number of pairs of successive NNs that differ by more than 50 ms (NN50), and percentage of NN50 (pNN50) [39]. The frequency domain of HRV included very low frequency (VLF; 0.0033–0.04 Hz), low frequency (LF; 0.04–0.15 Hz, refers to sympathetic and parasympathetic nervous systems co-regulation or baroreceptor gain), high frequency (HF; 0.15–0.40 Hz, refers to the activity of the parasympathetic nervous system), total power (TP; 0.0033–0.4 Hz, refers to total HRV), and LF/HF ratio (refers to the activity of the parasympathetic nervous system) [39]. Due to the skewed HRV distributions, VLF, LF, HF, and TP of HRV were transformed using natural logarithms into lnVLF, lnLF, lnHF, and lnTP.

To investigate which multiple risk factors could predict HbA1c values and determine whether machine learning methods were suitable for accurately classifying poor and normal glycemic control groups, the study used the following statistical analyses. Firstly, regression tree analysis was conducted, which has been used in previous studies to identify risk factors that predict health outcomes [40,41,42]. In this study, we used regression tree analysis to determine which multiple risk factors can lead to high values of HbA1c. Secondly, while the previous analysis showed how different factors interplayed to result in high HbA1c values, it did not suggest how accurately these factors can predict the probability of developing T2DM, nor which machine learning method is best for this classification task. To address this gap, we compared different machine learning classification approaches to determine the most useful method for identifying poor or normal glycemic control groups. We transformed the HbA1c outcome variable using the criteria of 6.5% [43] and divided it into two groups: the poor glycemic control group (HbA1c values ≥ 6.5%, *n* = 495) and the normal glycemic control group (HbA1c values < 6.5%, *n* = 152).

To determine the best machine learning method for accurately classifying poor or normal glycemic control groups, we compared several classification techniques, including support vector machine (SVM), boosting, classification tree, neural network, K-nearest neighbors, and random forest. To avoid biased estimation due to unequal class sizes between groups [44], we balanced the class sizes using the groupdata2 package in R [45,46] for oversampling the minority group (which is the normal glycemic group, resulting in a final sample size of 990 subjects, with 495 in each group). We randomly split the data into 80% training data and 20% testing data and coded the outcome variable as 1 for the poor glycemic control group and 0 for the normal glycemic control group.

To compare the models, we examined their sensitivity, specificity, area under curves, and classification accuracy rate with the testing data. Sensitivity represents the proportion of correctly classified positive cases, while specificity represents the proportion of correctly classified negative cases. The AUC is the area under the receiver operating characteristic curve, which plots sensitivity against 1-specificity. We used the R packages rpart [47] and machine learning functions in JASP [48] to perform the above analyses.

## 3. Results

### 3.1. Regression Tree

The regression tree analysis used the full 647 samples. In classification tree analysis, the balanced sample was the normal glycemic control group (*n* = 495; 50%) and the poor glycemic control group (*n* = 495; 50%). The regression tree analysis identified multiple risk factors that could lead to higher values of HbA1c. The optimal fit of the model was achieved using the complexity parameter (cp) of 0.01. The starting node indicated that the average HbA1c value of the group was 7.2. The analysis suggested that patients with multiple risk factors had higher HbA1c values than those with only one risk factor. The following were the key findings:

Group 1: Patients with SDNN < 14, BMI < 34, age < 54 years old, and lnVLF < 2.6 had the highest HbA1c values (11%).

Group 2: Patients with SDNN < 14 and BMI ≥ 34 had high HbA1c values (9.5%), although it was less than the first group.

Group 3: Patients with almost the same conditions as the first group but with lnVLF ≥ 2.6 had less high HbA1c values (7.9%).

Group 4: Patients with SDNN < 14, BMI < 34, age ≥ 54 years old, and LDL ≥ 107 had higher HbA1c values (8.6%).

Group 5: Patients with multiple risk factors, including SDNN < 14, 25 ≤ BMI < 34, age ≥ 54 years old, LDL < 107, and HDL < 37 had higher HbA1c values (8.3%).

Group 6: Patients with SDNN ≥ 14, TG ≥ 72, LDL ≥ 145 had high HbA1c values (8.1%).

Group 7: Patients with SDNN ≥ 14, TG ≥ 72, LDL < 145, PHQ-9 score ≥ 3, and BMI ≥ 23 had HbA1c values of 7.5%.

**Figure 1:**
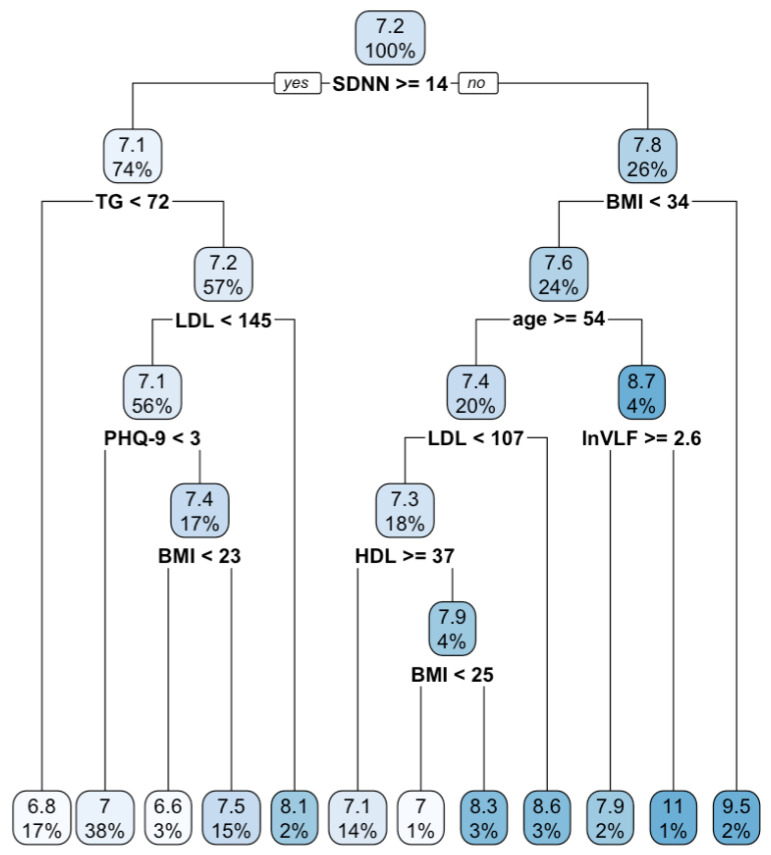
Regression tree for type II diabetes (N = 647).

The analysis suggested that multiple risk factors interacted with each other to result in higher HbA1c values. The results showed that one risk factor alone did not necessarily lead to higher HbA1c values. Figure 1 displays the regression tree results.

Note: BMI, body mass index; HDL, high-density lipoprotein; LDL, low-density lipoprotein; lnVLF, natural logarithms of very low frequency; PHQ-9, Patient Health Questionnaire-9; SDNN, the standard deviation of normal-to-normal intervals; TG, triglycerides. In each node, the number denotes the predictive mean HbA1c value of individuals within that group, while the percentage represents the proportion of individuals in that node relative to the total sample size.

### 3.2. Comparisons of AI Machine Learning Classification Methods

Table 1 summarizes the comparison of various machine learning classification methods including SVM, boosting, classification tree, neural network, K-nearest neighbors (KNN), and random forest, in terms of sensitivity, specificity, the area under curves, and accuracy rate on testing data. Our analysis showed that random forest had the highest sensitivity (77%) and specificity (91%), resulting in an accuracy rate of 84% and the largest area under curves of 95% among all the machine learning methods (Table 1).

## 4. Discussion

A gap in the previous literature was the limited inclusion of depression and anxiety as predictors of diabetes. In this study, we found evidence suggesting that depression can be an important factor in certain subgroups of T2DM. Although the causality between depression and diabetes is not clear, previous studies have reported a high comorbidity rate between these two diseases [49,50]. While some studies have examined depression, they either used depression as the outcome in a group of T2DM patients or as a predictor of other co-occurring diseases in patients with T2DM. The current study has included depression and anxiety to examine their relations with diabetes directly. Using regression tree analysis, this study identified three pathways of multiple risk factors associated with poor glycemic control in T2DM patients who have low parasympathetic activation and whose age is younger than 56 years old or in patients who have low parasympathetic activation (HF), whose age is higher than 56 years old with high LDL, and whose LF is higher or lower than 1.2.

The results of the regression tree in this study provide valuable information on how multiple factors interact to create subgroups within diabetes patients and can be informative for developing prevention strategies for T2DM. Traditional regression analysis is useful in identifying risk factors [51] but does not provide information on how these factors interact. In contrast, the regression tree analysis used in this study revealed different sets of conditions that could all lead to high HbA1c values. This approach is particularly useful in situations where people may not have certain critical risk factors but still have high HbA1c values. While some recent studies have applied decision tree analysis to understand subgroups in diabetes-related situations [25,26], the focus of these studies was not on how risk factors interact to create subgroups for HbA1c values. The current study aims to fill this gap in the literature by providing information about subgroups that can be informative for clinicians. The use of regression tree analysis is a valuable methodological contribution, as it provides a more nuanced understanding of the complex relationships between multiple risk factors and poor glycemic control in T2DM patients.

The second analysis aimed to provide further insight into the comparison of different machine learning methods for predicting diabetes, as the results from previous studies were diverse [21,22]. Our classification analysis showed that random forest had the highest prediction rate (84%) for the outcome of T2DM, among several machine learning methods. This finding is consistent with previous studies that also found random forest to be the best model [9,10,13,15], while others did not [11,12,14]. In our study, we included variables such as depression and anxiety that were not analyzed in previous studies. This could be one of the reasons why SVM was not the best-performing method in our analysis, as some studies have suggested [21]. However, a review by Abhari et al. [22] pointed out that it is important to note that the performance of different machine learning methods can vary depending on the variables being included, the methods being used, and the types of outcomes being analyzed. Therefore, our findings should be interpreted in the context of our study design and variables. In addition, the study by Raghavendra and Santosh [52] suggests another possible explanation for our finding that random forest outperformed SVM in our analysis. They found that random forest performs better than other machine learning methods when studies have fewer variables. This may be the case in our study. Therefore, the complexity of our dataset may have contributed to the superior performance of random forest over SVM.

This study has several limitations that should be considered when interpreting the results. First, the sample size of 647 patients with T2DM may be considered small for artificial intelligence machine learning analysis. Although we used balanced methods to increase the sample size of the normal glucose group to have an equal class size, the sample size was only up to 990. Previous studies have shown that machine learning can still be potentially biased with a sample size of fewer than 1000. Therefore, future studies should aim to increase the sample size to confirm the risk factors for T2DM identified in the regression tree model. Second, the data on blood glucose and lipid profiles were collected only once, which may not fully represent the long-term glycemic control for patients with T2DM. Collecting blood samples multiple times over a long period of time and exploring possible risk factors for poor glycemic control may provide more accurate results. Third, while we found that depression is a risk factor for a subgroup of T2DM patients, the causality between depression and diabetes is still not fully clear. Despite these limitations, our comparison of machine learning methods suggested that random forest is a more accurate method for analyzing small sets of features and is particularly useful when depression is included as a risk factor. Further research can extend these findings by using cross-lagging models with longitudinal data to better understand the relationship between depression and diabetes.

## 5. Conclusions

In conclusion, our study identified key risk factors and pathways for poor glycemic control in patients with T2DM using the regression tree algorithm. The random forest approach found that multiple risk factors are important in screening, monitoring, diagnosis, and prevention of poor glycemic control, such as considering demographic data, physiology dimensions (BMI, lipid profiles, and HRV indices), and emotional dimensions (depression and anxiety). The multiple risk factors can also be considered as a framework for designing potential intervention programs in future studies.

## Figures and Tables

**Table 1:** Comparison of classification methods of AI machine learning.

Model	Testing Data			
%	Sensitivity	Specificity	Area under Curves	Accuracy Rate
Boosting	51%	63%	63%	57%
Support vector machine	52%	65%	58%	58%
Classification tree	59%	80%	69%	69%
Neural network	69%	80%	60%	74%
K-nearest neighbors	63%	93%	78%	78%
Random forest	77%	91%	95%	84%

## Data Availability

Data are available via correspondence upon request.
